# Supplementary material for: Are carbon emissions trading and green financial instruments synergistic? -Comprehensive quantitative research based on content analysis
Source: PLoS One. 2024 Mar 7;19(3):e0298601. doi: 10.1371/journal.pone.0298601 (PMC10919601; doi:10.1371/journal.pone.0298601)
Supplement: S1 Data — (ZIP) [file pone.0298601.s001.zip › Data/地级市Z_F工作报告.zip!/╕ú╜¿/╧├├┼╩╨/└┤╫╘╣1⁄2╓┌║┼í╛┬φ┐╦╩2╛▌═°í┐_2012.docx]

各位代表:

　　现在，我代表市人民政府向大会作政府工作报告，请予审议，并请市政协各位委员和其他列席人员提出意见。

　　一、2011年和过去五年的工作回顾

　　2011年，我们在市委的坚强领导下，高举中国特色社会主义伟大旗帜，以邓小平理论和“三个代表”重要思想为指导，深入贯彻落实科学发展观，扎实推进科学发展新跨越，完成了市十三届人大六次会议确定的各项任务，实现“十二五”良好开局。预计全年地区生产总值2527亿元，增长15%；规模以上工业总产值4460亿元，增长18.3%；全社会固定资产投资1125亿元，增长30.1%；财政总收入652亿元，增长26.2%，其中地方级财政收入371亿元，增长32.6%；社会消费品零售总额800亿元，增长16%；城镇居民人均可支配收入33485元，增长14.5%，农民人均纯收入11910元，增长18.7%；城镇登记失业率3.45%，居民消费价格指数105，人口自然增长率9.38‰。完成省下达的节能减排目标任务。“五大战役”总体进度居全省领先。

　　这一年，我市实现全国文明城市“三连冠”，深化两岸交流合作综合配套改革试验总体方案获国务院批准，荣膺全国十大创新型城市和十大低碳城市，位居全国科学发展典范城市综合排名前列，名列中国服务型政府十佳城市前茅，以优异成绩庆祝厦门经济特区建设30周年。

　　一年来，我们主要做了以下工作:

　　（一）进一步提高产业素质。围绕壮大产业集群，出台制造业产业集聚发展的实施意见。全年完成工业投资230亿元，增长11%。平板显示产业链实现产值近千亿元。天马面板等一批项目开工建设，太古飞机六期等一批项目竣工投产。全年规模以上工业增加值增长19.4%，工业利润增长17.6%，工业经济效益综合指数达210。新增中国驰名商标12件，总量达51件。

　　围绕建设国家创新型城市，不断增强自主创新能力，国内发明专利授权551件，增长35%。新增4个国家级标委会，主导和参与制定30项国家标准。新认定高新技术企业153家，福建中烟技术中心成为国家级企业技术中心，特宝生物列入国家地方联合工程研究中心发展计划，新增通士达等4家国家创新型企业，高新技术企业产值增长33%。厦门大学“重组戊肝疫苗”获批国家一类新药。火炬高新区列入国家创新型科技园区试点，厦门科技创新园动工建设。“十城万盏”任务全面完成。

　　围绕提升现代服务业，积极培育壮大支柱产业。厦门港货物吞吐量达1.56亿吨，增长12.2%；集装箱吞吐量642万标箱，增长10.2%。建成海沧6个十万吨级泊位，新增集装箱吞吐能力390万标箱。空港旅客吞吐量1570万人次，增长19%，开通厦门至阿姆斯特丹洲际空中客运航线。举办会议2200场，展览活动150场。接待境内外游客3510万人次，增长16%。新增6家高星级酒店。“海洋神话号”邮轮首航台湾、香港。两岸区域性金融服务中心纳入国家“十二五”规划，并动工建设。

　　浦发银行、国家开发银行、中国进出口银行在厦设立分行，新增上市企业5家，新增金融保险证券机构11家。湖里万达、海沧阿罗海城市广场等建成投入使用。戴尔服务（中国）等一批总部落户厦门，离岸服务外包合同金额和执行金额分别增长80%和86%。新认定软件企业70家，软件和信息服务业销售收入350亿元，增长35%。

　　（二）进一步加快岛内外一体化进程。加快东渡港区物流功能调整，启动邮轮母港规划。鼓浪屿申遗工作进展顺利，整治工作初见成效。旧村旧城改造力度加大。沿街建筑立面整治、夜景亮化等工程有效推进。完成主要道路绿化提升，4.5公里长的老铁路改造成文化公园。枋湖客运中心、第一码头公交枢纽站建成投入使用。

　　加快岛外四个新城建设，完成总投资305亿元。一批标志性工程有序推进，一批市政设施基本完工，一批公建项目加快建设。穿越岛外四区的海翔大道一期工程38公里基本建成，成为重要疏港通道。集杏海堤开口改造竣工，海域清淤工程加快推进。高崎机场三期扩建正式动工，翔安国际新机场进入国家报批程序。四个试点小城镇改革建设全面展开，完成总投资100亿元。

　　厦漳泉同城化合作框架协议正式签署，推进首批18个重大项目建设。厦漳跨海大桥、厦安高速、厦成高速等一批同城化重点项目顺利推进，九龙江北溪引水左干渠二期改造工程基本建成，向厦门供水的长泰枋洋水库获国家批准。

　　（三）进一步提高改革开放水平。整合组建信息、金圆、轨道、市政四家国有企业集团。统一城乡居民基本医疗保险政策，筹资标准提高到每人每年380元，住院医疗费统筹基金支付比例提高到70%，惠及城乡居民90多万人。开展公交票制改革，进一步降低票价。创新社会管理体制机制，实行流动人口“一站式”服务，实现流动人口、出租房屋登记全覆盖。整顿提升民办幼儿园质量，加大财政补助，较大幅度缩小民办与公办幼儿园的收费差距。推进公立医院改革，成立我省首家中外共建的厦门——新加坡友好医疗服务中心。厦门被评为全国文化体制改革工作先进地区。

　　进一步放宽视野、拓展领域，加大“三维”招商力度。对接央企项目22个，总投资1755亿元；民企项目163个，总投资1590亿元；新设立外商投资项目360个，实际利用外资22.6亿美元，千万美元以上项目合同外资占全市总量的77%。成功举办第15届投洽会，高端论坛和专业展会更加丰富，实现内涵提升、外延拓展。增加节能灯、食用菌两个国家首批外贸转型升级示范基地，入选国家船舶出口基地。全年外贸进出口698亿美元，增长22.4%。积极开展区域协作，完成援助南平灾后重建，对口支援西藏、新疆、宁夏等地区。

　　（四）进一步深化两岸交流合作。成功举办第三届海峡论坛，办好台湾特色庙会、郑成功文化节等配套活动，凸显民间性、大众性和广泛性。台交会、文博会、图交会等展会影响力不断扩大。国家部委在厦设立海峡两岸教育交流与合作基地、海峡两岸传媒人才交流与培训中心、青礁白礁慈济宫海峡两岸交流基地。开通厦门直航台南空中航线。厦门居民赴台个人游、省外来厦暂住人员赴金门一日游正式启动。厦金航线运送旅客近140万人次，增长9%；经厦门口岸赴台湾旅游20万人次，增长21%。加强与台湾产业对接，两岸现代服务业合作区、海峡旅游服务中心等项目顺利推进，建成对台邮件处理中心。率先落实《海峡两岸经济合作框架协议》。全年对台进出口总额72亿美元，增长13%。

　　（五）进一步提高“三农”工作水平。认真落实支农强农惠农政策，农民人均纯收入增幅首次超过城镇居民人均可支配收入。全年实现转移就业2万多人。完成30个社区集体资产改制。新增2家国家级和9家省级农业产业化龙头企业，农副产品与食品加工业实现产值300亿元，增长24%。都市型现代农业快速发展，四个村镇获评全国“一村一品”示范村镇。加大农田水利基础设施建设，开工建设莲花水库，加快石兜水库除险加固等水利工程。启动汀溪上游5个行政村的连片整治工作，“家园清洁行动”成效明显。实施农村客运公交化工程，有线电视入户率达98.5%，农村自来水管网改造基本完成。

　　（六）进一步加快社会事业发展。实现城乡义务教育完全免费，对中等职业学校一年级学生免除学费。进城务工人员随迁子女就读公办义务教育学校达10.5万人。新建、扩建23所中小学校，义务教育学校标准化建设比例达92%。新增公办幼儿园19所。加固中小学校舍93幢。全面建成中小学校与公安110联网的监控报警系统。顺利通过国家卫生城市复查。第一医院门急诊综合楼竣工，眼科中心扩建工程加快推进。城市居民健康档案建档率达79.5%。持续稳定低生育水平，出生人口政策符合率96.22%。成功举办第三届中国诗歌节、国际戏剧协会第33届世界代表大会。推进博物馆、美术馆、公共图书馆免费开放。杏林湾皮划赛艇训练基地投入使用。厦门国际马拉松赛连续四年获“国际田联金牌赛事”称号。双拥共建、外事侨务、人防海防、地震气象、方志档案、民族宗教、妇女儿童、老龄、残疾人等工作取得新成绩。

　　（七）进一步提升民生保障水平。集中力量办好十大民生工程和30项惠民实事。企业退休金增至人均每月2013元。重度残疾人享受城乡居民养老待遇的年龄提前到55周岁。开工建设4.1万套保障房，配租配售5320套。新开通公交线路77条，新增与更新公交车596辆，新建256座公交候车亭、4个公共停车楼和149座公厕。新增130个社区居家养老服务中心。通过定向供应平价农副产品，平抑市场物价。继续提高城乡低保对象的补助标准，发放动态价格补贴，保障低收入群体基本生活。治理餐桌污染，加大农产品和食品生产、流通等环节的监管力度。造林绿化2万多亩，新增和改造提升园林绿地1.6万亩，空气质量优良率99.4%。建成机动车排气检测中心。加强地质灾害防治，建成742个自然灾害避灾点，实施农村地质灾害点移民造福工程。推进平安厦门建设，完善社会矛盾纠纷排查调处机制。落实安全生产责任制，有效减少安全生产事故。

　　（八）进一步加强政府自身建设。加快政府职能转变，落实一岗双责，强化监察与审计工作，廉政建设得到进一步加强。规范权力运行，清理行政审批项目，取消、调整、下放幅度达45%。扩大网上审批、并联审批，行政审批再次提速。完善政府采购制度，扩大公共资源市场化配置范围。健全绩效评估体系，开展治庸治懒治散专项行动。加强民主法制建设，清理政府规章和规范性文件，废止或修订政府规章16件、规范性文件31件；提请市人大审议法规草案7件，制定政府规章5件、规范性文件68件。依法清理行政强制主体，加强对重大行政处理、重大行政处罚和委托执法的备案审查，提升行政执法能力。设立行政复议委员会，试行行政复议相对集中审理。坚持依法向市人大及其常委会报告工作，认真执行市人大及其常委会决定决议。自觉接受市政协民主监督，认真听取民主党派、工商联、无党派人士、人民团体和社会各界的意见和建议。全年办理人大议案7件、建议215件，满意率97.2%；办理政协提案507件，满意率96.3%。

　　各位代表！2011年各项任务的完成，为本届政府的任期目标画上了圆满句号。过去的五年，全市人民团结奋进，求真务实，有效应对国际金融危机冲击，战胜前进中遇到的各种困难和风险，是经济建设取得重大进展的五年，是城市综合竞争力显著增强的五年，是朝着现代化国际性城市迈进的五年，是对台交流合作不断拓展的五年，是社会事业民生保障明显提升的五年。

　　五年来，经济实力持续壮大。较好实现了发展速度和质量效益的同步增长，2011年与2006年相比，地区生产总值由1174亿元提高到2527亿元，规模以上工业总产值由2368亿元提高到4460亿元，按可比价格计算，分别增长90%、108%；财政总收入由275亿元提高到652亿元，地方级财政收入由144亿元提高到371亿元，按可比口径计算，分别增长148%和182%。全市高新技术企业665家，高新技术企业产值占全市规模以上工业总产值比重达40%，万元生产总值能耗约为全国平均值一半，超额完成节能减排目标，被授予全国“节水型城市”和“节能减排二十佳城市”，成为国家创新型城市试点。社会消费品零售总额年均增长18.3%；航运物流、旅游会展、金融商务、软件信息成为支柱产业，推进现代服务业快速发展。

　　五年来，城市承载力持续提升。累计完成固定资产投资4876亿元，城市建成区扩大近一倍，岛外四个新城建设全面展开。建成翔安海底隧道、集美大桥、杏林大桥、城市快速公交、成功大道、环岛干道等一批重大交通基础设施，城市快速交通网络基本形成。厦漳港口实现一体化，集装箱吞吐量升至全球第19位，福厦高铁通车，厦门北站投入使用，厦门航空港成为我国第五大口岸机场。建成城市液化天然气管道、东部供水工程等一批重大市政项目。城市现代化信息化水平进一步提高。

　　五年来，发展动力持续增强。经济特区范围扩大到全市。国家服务业综合改革试点城市、全国保险改革发展试验区、服务外包示范城市、“三网融合”试点城市、低碳城市试点等改革扎实推进。统筹城乡发展、文化体制、医药卫生体制改革取得成效。海沧保税港区首期封关运作，二期通过预验收。国有企业净资产由480亿元增加到1070亿元，增长123%。累计实际利用外资84亿美元，新增世界500强企业投资项目18个。外贸进出口规模占全省一半以上，成为跨境人民币结算试点城市。国际友城增至15个，友好港口达19个。

　　五年来，对台优势持续凸显。率先成为两岸航空客运直航点、海上直航口岸和通邮封发局，开通两岸客滚轮定期班轮。五年实际利用台资27亿美元，对台进出口贸易总额246亿美元。一批台资金融机构入驻厦门。大陆最大的台资合资医疗机构——长庚医院开业。厦门成为大陆最大的台湾水果集散中心和首批两岸食品物流合作试点城市。国家部委在厦设立了20多个对台交流合作基地，海峡论坛等涉台盛会影响力持续扩大。

　　五年来，社会和谐程度持续提高。全市财政一般预算60%以上投入民生和社会事业。新增就业近百万人。全面完成农村义务教育和公共卫生体系建设，义务教育均衡发展。医疗资源总量提高，新增6家三甲医院，每千人卫生技术人员达5.15人，每千人病床数达3.15张。建立覆盖全体居民的城乡一体化社会保障体系，给60岁以上老人每月发放200元基础养老金。基本建立覆盖中低收入家庭的住房保障体系，建成2.3万套保障性住房，实现低保家庭廉租住房应保尽保。农民人均纯收入持续位居全省首位。

　　五年来所取得的成绩，是在党中央国务院、省委省政府和市委的正确领导下，在市人大与人民代表的依法监督下，在市政协与全体委员的民主监督下，全市广大干部群众齐心协力、共同奋斗的结果。在此，我谨代表市人民政府，向全市人民，向各民主党派和工商联、人民团体、各界爱国人士，向离退休的老同志，向中央和省驻厦单位，向驻厦人民解放军、武警官兵和公安干警，向来厦的所有投资者和劳动者，向所有关心支持厦门的港澳同胞、台湾同胞、海外侨胞和国际友人，表示衷心的感谢和崇高的敬意！

　　各位代表！

　　五年的工作让我们深深体会到:必须坚持把握经济特区发展规律，科学定位城市发展，体现厦门特色和时代特征，积极探索符合厦门实际的现代化国际性港口风景旅游城市发展道路。必须坚持改革创新，弘扬先行先试、敢为人先的特区精神，用改革创新的办法，突破体制瓶颈，破解发展难题，为特区永续发展不断注入新的活力和动力。必须坚持走生态文明之路，建设好、保护好厦门优美宜居的自然环境和人文环境，不断提升厦门宜居水平和环境竞争力。必须坚持发挥对台前沿平台优势，时刻牢记经济特区特殊使命，不断推动两岸交流合作迈向多层次、宽领域、常态化。必须坚持把保障和改善民生作为政府一切工作的出发点和落脚点，积极主动创造条件，切实解决人民群众最关心、最直接、最现实的利益问题。

　　同时，我们也清醒地看到，经济社会发展和政府工作还存在许多困难和问题，主要是:经济发展的不确定因素和困难因素不断增加，国际经济形势复杂严峻，国内经济增长下行与物价上涨压力相互交织；经济转型、产业结构调整和发展方式转变的任务更为艰巨，经济抗风险能力比较脆弱，资源和环境压力不断增大；改善民生、促进和谐的任务依然繁重，城市管理、社会管理中群众关心的热点难点问题，特别是岛内交通拥堵和停车难问题还没有得到很好解决；制约科学发展的体制机制问题仍然存在；一些政府工作人员的服务意识不强、办事效率不高。对此，我们必须高度重视，在今后工作中切实加以克服和解决。

　　二、今后五年工作展望和2012年工作建议

　　在厦门经济特区建设30周年庆祝大会上，党中央国务院对厦门提出了殷切期望，赋予新的历史使命。市第十一次党代会深刻分析了今后五年面临的机遇和挑战，作出了立足新起点、当好排头兵，奋力推进厦门科学发展新跨越的战略部署。我们要全面贯彻落实，牢牢把握历史机遇，扎实推进各项工作，努力实现经济社会又好又快发展。

　　今后五年，政府工作总体要求是:高举中国特色社会主义伟大旗帜，以邓小平理论和“三个代表”重要思想为指导，深入贯彻落实科学发展观，在党中央国务院、省委省政府和市委的坚强领导下，突出主题主线，继续解放思想，深化改革开放，按照全省科学发展、跨越发展的战略部署，全面加快岛内外一体化和厦漳泉同城化，全力打造创新厦门、宜居厦门、平安厦门、文明厦门、幸福厦门，在新的起点上推进科学发展新跨越，努力当好推动科学发展、促进社会和谐、服务和平统一的排头兵，更好更快地建设现代化国际性港口风景旅游城市和海峡西岸重要中心城市。

　　根据这个要求，我们要确保全面完成“十二五”规划目标，努力实现六个新跨越:

　　——改革开放实现新跨越。不失时机地推进重要领域和关键环节改革，落实综合配套改革试验总体方案，加快建设国家创新型城市、服务业综合改革、义务教育均衡发展、公立医院改革等试点。实行更加积极主动的开放战略，坚持“引进来”和“走出去”并重，有效利用国际国内两个市场、两种资源，拓展新的开放领域和空间，全面提升开放型经济水平，增创经济全球化条件下参与国际经济合作和竞争新优势。

　　——经济发展实现新跨越。在优化结构、提高效益、降低能耗、保护环境的基础上，实现高新技术产业产值、财政总收入、地方级财政收入、社会消费品零售总额、民生和社会事业总投入“五个翻番”。高新技术产业和新兴产业产值占规模以上工业总产值比重持续提升，科技进步贡献率不断提高。打造具有国际影响的千亿以上产值的产业集群，建设海西强大的先进制造业基地、领先发展的新兴产业示范区。

　　——中心城市建设实现新跨越。打造现代服务业集聚区，持续壮大航运物流、旅游会展、金融商务、软件信息等支柱产业。加快建设东南国际航运中心，港口货物吞吐量超过两亿吨、集装箱吞吐量突破千万标箱。加快建设国际邮轮母港，构筑高端邮轮经济商业圈。开工建设翔安国际机场、城市轨道交通，建成厦深、龙厦高铁等重要交通设施。加快厦漳泉同城化步伐，打造厦漳泉大都市区。岛外新城核心区基本建成，城市建成区面积超过300平方公里，城市化率达85%，海峡西岸重要中心城市集聚辐射能力进一步提升。

　　——对台交流合作实现新跨越。着力构建两岸经贸合作最紧密区域、两岸直接往来最便捷通道、两岸交流交往最活跃平台、两岸同胞融合最温馨家园，把厦门的优势和特色体现在与台湾的经济合作和贸易往来上，体现在发挥桥梁纽带作用上。进一步加强人员往来、扩大各界交流、增进相互了解；进一步维护台湾同胞正当权益，满腔热情为台湾同胞多办好事、实事，努力为推动两岸关系和平发展作出新贡献。

　　——生态文明建设实现新跨越。创建国家低碳城市，单位生产总值能耗、碳排放强度、主要污染物排放量等重要指标居全国领先水平。创建国家森林城市、国家生态园林城市和国家环保模范城市，城市建成区绿化覆盖率达42%以上，人均公共绿地达11.5平方米以上。加大环境综合整治与生态修复，提高城市空气质量，努力向无尘化目标迈进。

　　——人民生活水平实现新跨越。坚持基本公共服务均等化，维护公平正义、体现公平正义。继续提高社会保障和基本公共服务水平，实现学有所教、劳有所得、病有所医、老有所养、住有所居。坚持教育优先，推动文化发展，全面提高市民道德文化素质，促进就业机会更加均等。保持城乡居民收入居全国前列，城乡居民主要健康指数基本达到发达国家水平，社区居家养老服务网络实现全覆盖。建立政府为主导的基本住房保障制度，城市公共文明指数和幸福指数明显提升。

　　各位代表！

　　2012年是新一届政府履职的第一年。我们要充分认识当前复杂严峻的经济形势，保持清醒头脑，做好充分的思想准备和应对措施，全面贯彻落实中央经济工作会议精神，按照省委省政府和市委的决策部署，激发工作热情，明确目标任务，采取得力措施，抢抓有利机遇，克服各种困难，确保稳中求进，全力以赴开好局，起好步。

　　经济社会发展主要预期目标是:地区生产总值增长12%，规模以上工业总产值增长12%，全社会固定资产投资增长18%，外贸进出口增长12%，实际利用外资22亿美元，财政总收入和地方级收入增长15%，城镇居民人均可支配收入和农民人均纯收入增长12%，城镇登记失业率控制在4.2%以内，居民消费价格指数104左右，人口自然增长率控制在10‰以内。完成国家和省下达的节能减排指标任务。在实际工作中要加倍努力，力争完成得更好。

　　为此，我们要扎实做好以下工作:

　　（一）坚持优化结构，加快产业发展转型升级

　　大力提升产业功能区规模集聚效应。一是集美机械工业园、同安工业集中区等工业产业功能区，要完善生产生活配套设施，着力延伸产业链，壮大产业群，提升产业层次。二是火炬（翔安）产业区、科技创新园、软件园二期、海沧生物医药园等科技产业功能区，要大力发展高新技术产业、战略性新兴产业。三是两岸区域性金融服务中心、现代物流园区、海沧保税港区、观音山、五缘湾、杏林湾商务营运中心等现代服务业功能区，要加快集聚高端服务业项目。四是加强海洋自然保护区和海洋生态功能区建设，大力发展海洋经济，重点发展海洋资源综合利用，加快海洋生物科研成果产业化，推动厦门国家海洋研究中心建设。五是规划建设一批新的产业功能区，加快推进软件园三期和火炬高新区扩区建设，吸引一批大项目落地。

　　大力提升先进制造业发展水平。围绕“抓龙头，铸链条”，进一步做强做大电子、机械等支柱产业，巩固提升视听通讯、钨材料等五个国家特色产业基地，持续壮大光电显示产业，积极推进国家大型邮轮建造基地落户厦门，做好国家海洋与生命科学产业集群试点工作。改造提升传统优势产业，增强企业新产品开发能力和品牌创新能力。建成一批高新技术产业项目，培育一批战略性新兴产业，确保一批重点工业项目如期开工。办好首届中国工业设计大赛，推进工业设计发展。加快信息化与工业化的融合。抓好国家级循环经济标准化试点建设，突出重点领域的节能减排，强化监督管理，落实目标责任。

　　大力提升现代服务业辐射功能。重点发展航运物流，拓展国际中转、内支线中转、海铁联运等业务，加快“陆地港”建设，做大做强厦门石油交易中心。加快发展金融商务，积极引进各类金融机构。继续争取火炬高新区进入“新三板”扩容试点，培育更多的企业上市。大力发展电子商务。突出发展旅游会展，推动闽南古镇、五缘湾游艇帆船国际展销中心、直升机旅游服务中心建设。加快建设环东海域休闲酒店群和文化产业园，整合提升文化旅游景区建设。支持品牌展会，建成会展中心三期，扩大投洽会的全球影响力。着力发展软件信息产业，加快推进云计算应用与产业发展。继续建好“无线城市”和“宽带城市”，支持建立“物联网产业联盟”，保持厦门在全国移动互联网应用的领先地位。

　　大力提升自主创新能力。整合研发资源，形成层次丰富、结构合理的产业研发布局。更好发挥厦门产业技术研究院作用，积极引进中科院、央企、跨国公司、重点大学等在厦设立研发机构，推进中国电子科技集团三十所、中船重工七二五所等在厦研发机构建设，与海西研究院共建稀土研究所。深化创新型企业示范和试点工作，支持企业建立国家工程技术研究中心、重点实验室和重大科技创新平台。积极推进国家知识产权工作示范城市和国家专利产业（厦门）试点基地建设，提高知识产权创造、运用、保护和管理水平。高度重视人才引进和开发，加强高技能人才培养，落实“双百计划”，吸引更多海内外高层次人才来厦创业。

　　大力帮扶企业增强竞争力。一是加大政银企对接力度，帮助企业缓解融资难。落实好支持小微企业发展的政策措施，设立中小企业信用担保机构贷款风险补偿专项资金，规范发展小额融资贷款公司。二是帮助企业开拓市场。发挥商品展销会等平台作用，支持企业扩大进出口，拓展内销市场。三是提高服务企业的水平。建设中小企业公共服务平台。实行“一企一策”帮扶措施，推动企业转型升级。加大财政扶持力度，阶段性降低部分涉企收费，减轻企业负担。

　　（二）坚持拓展提升，提高城市发展水平

　　着力拓展岛外。重点是拓展空间、集聚产业、提升功能。按照一流标准加快建设集美、海沧、同安、翔安新城核心区，形成各具特色、功能突出的新城格局。加快前场铁路大型货场、厦门北站动车运用所等项目建设，形成东南沿海铁路主枢纽。优化港区功能布局，推进码头资源整合，完成海沧航道三期扩建，支持中远集团建设远海港区装卸设备自动化工程，提高港口信息化、自动化水平，加快建设东南国际航运中心。抓紧翔安国际新机场各项报批与规划设计工作，开工建设机场高速公路，抓紧与漳泉两市快速路网的对接。同时，加快新城之间路网建设，实施同集路改造提升，完成海翔大道二期工程建设。加快同安抽水蓄能电站项目前期工作，力争早日开工。

　　着力提升岛内。重点是形象更新、产业转型、功能完善。加快建设国际邮轮母港，实现航线常态化运营。启动五缘国际高端医疗园整体规划，推动五缘综合医院、国际肿瘤专科医院等项目动工。加快筹建世界名牌购物中心。强化鼓浪屿保护管理，按照“遵循规划，抓住重点，整体推进，务求实效”的原则，扎实做好核心区56个要素的恢复整治，全力冲刺2013年世界文化遗产申报。加快推进旧城镇、旧村庄、旧厂房改造，推进厦港、将军祠、后埔枋湖、何厝岭兜等片区提升改造，鼓励工业企业加速向岛外搬迁。加快推进会展北、湖边水库、五缘湾等片区建设，打造具有国际水准的东部新城区。

　　提升城市管理水平。加强城乡规划，强化土地资源集约利用。完善城市管理长效机制，提升城市管理的科学化水平。推进数字厦门建设，构建统一的地理信息公共平台。新建主干道路必须同步建设共同管沟，实现缆线下地。继续强化夜景工程、绿化美化工作。提高交通智能化水平，加快交通“绿波带”建设。开工建设轨道交通一号线，打通一批断头路，推进慢行系统建设。加强电动车的规范管理。着力解决停车难问题。变城市道路保洁清扫为清洗，管好土方车，治理汽车尾气，提高城市空气质量，推进绿色社区和安静小区建设。加快海域清淤整治，完善城乡污水垃圾处理等市政设施，加快建设重点节能减排项目，全面建设生态城市。

　　加快厦漳泉同城化。全力推进已确定的项目，筛选并实施第二批同城化重点项目。完善交通基础设施网络，在时空上缩短距离，推动三市产业优势互补，实现公共资源、大型基础设施共享。开工建设长泰枋洋水库，从根本上保障厦门城市用水安全性、可靠性。

　　（三）坚持强农惠农，加快社会主义新农村建设

　　多渠道促进农民增收。完善城乡一体的就业创业服务体系，支持农村富余劳动力参加实用技能培训，公益性岗位优先招收本市农村劳动力。鼓励扶持失地失海农民渔民转岗就业、自主创业，重点扶持乡村旅游、商贸流通和社区服务等创业项目。扶持发展农业专业合作社。通过项目带动、资产量化、股份分红等方式持续增加农民收入。

　　鼓励农业产业化、基地化。支持农业龙头企业科技研发和品牌建设，培育一批企业集团，做强做大农副产品加工与食品加工产业集群。建设对台农业新技术、新品种引进合作交流基地和国家农业科技园。加快“菜篮子”基地建设，鼓励农超对接，保障市场供应。

　　加快城乡公共服务均等化。深入推进统筹城乡发展，积极引导优质公共服务资源向农村拓展，均衡发展城乡教育、卫生、文化、体育等社会事业。全面推进汀溪、新圩、灌口、东孚等试点小城镇改革建设。继续实施地质灾害点移民造福工程。加大水利设施建设力度，加快汀溪水库群及深青溪、过芸溪等流域整治。

　　（四）坚持先行先试，进一步深化改革扩大开放

　　扎实推进综合配套改革。围绕产业结构优化升级、两岸经贸合作、一体化和同城化、社会建设与管理等重要领域，研究制定改革创新具体方案，确保综合配套改革工作有序推进。继续深化行政管理体制、投融资体制改革，加快实施新一轮市区财政体制改革，分类推进事业单位、国有企业、医疗卫生等改革，加快服务业综合改革、农村综合改革。

　　加快转变外贸增长方式。推进外贸公共服务平台和出口示范基地建设，争取成为国际贸易中心城市试点，推动外贸企业向内外贸整合方向发展。更好地发挥海关特殊监管区功能，创新通关模式，促进加工贸易向产业链高端延伸。扩大原材料、资源性商品等大宗货物进口。鼓励企业“走出去”，积极开拓新兴市场，开展跨境投资、并购上市。

　　全力推进“三维”招商。加强与国家部委和央企、民企、外企对接，集中力量引进一批世界500强企业和行业龙头企业。强化产业链招商，细化重点产业集群发展规划，策划生成一批带动力强的好项目、大项目。创新招商模式，完善市区两级联动招商机制，鼓励现有企业增资扩产，扩大以商引商。跟踪落实好“9·8”投洽会、“6·18”项目成果交易会和“三维”招商等签约项目。同时，落实好中央新一轮扶贫计划，继续做好对口支援和帮扶西藏、新疆、宁夏等工作。

　　（五）坚持发挥优势，拓展对台交流合作

　　突出经贸合作。加快建设两岸区域性金融服务中心，争取率先开展两岸货币清算、对台离岸金融业务，深入推进两岸跨境人民币业务。加快建设两岸现代服务业合作区，积极参与两岸产业搭桥计划。争取更多先行先试内容列入《海峡两岸经济合作框架协议》补充协议。积极推动与台湾基隆、台中、高雄等港区的对接合作。

　　拓展“三通”效应。加快建设五通码头改扩建工程，进一步争取开放航权、优化航路、增辟航线、增加航班，打造两岸海空运输综合枢纽。做好厦金“小三通”、厦台邮轮和客货滚装航线的宣传推介，加强厦台旅游合作，共推双向旅游线路。建成厦金海底通信光缆工程，为两岸民众提供更便捷优惠的通邮通讯服务。

　　密切交流交往。办好第四届海峡论坛等重大涉台活动。推进与台湾市县政府、基层乡镇之间交流互动，进一步提升各领域交流合作水平。继续争取简化赴台审批手续，争取国家有关部委、两岸协商机构及台湾行业协会来厦设立办事机构，更好地发挥对台交流合作基地的平台载体作用。

　　（六）坚持统筹协调，繁荣各项社会事业

　　提升教育事业发展水平。落实中长期教育改革发展规划纲要，扎实推进义务教育均衡发展。加强德育教育，提高师资水平，提升教育质量。规范幼儿园办学，新建、扩建一批公办幼儿园和中小学校。全面完成校安工程，抓好校车安全工作。实现中等职业教育全部免费，加强职业教育实训基地建设。提升高等教育质量，推进重点学科和重点专业建设。加强校企服务平台建设，增强高校服务产业发展的能力。加快推进厦大翔安校区、厦门理工学院三期、城市职业学院集美分院等建设。

　　提升医疗卫生服务水平。加强医德医风建设，严禁不合理用药、不合理检查，构建以电子病历为核心的医院信息系统及居民健康档案，切实减轻群众看病负担。完善基层医疗卫生服务体系，推进村级卫生所标准化建设，建成若干家省级以上示范社区卫生服务中心。加快推进“名院、名科、名医”建设，创建全国“百家优质医院”和“优质护理服务示范医院”。坚持计划生育基本国策，稳定低生育水平，提高人口素质。

　　提升城市文化软实力。加强社会主义核心价值体系建设，巩固全国文明城市创建成果。加快国家公共文化服务体系示范区创建工作，统筹布局公共文化设施，建好闽南文化生态保护区，建成闽南戏剧院，加快筹建厦门大剧院和海峡图书发行物流中心。鼓励创作文化精品，满足人民群众日益增长的精神文化需求。加强华文教育基地建设，传播弘扬中华文化。开发利用好史志、档案资源，完成第二轮地方志编修。依托大型文化产业项目，建设好文化园区，打造文化产业基地。加快发展创意设计、影视动画、文化旅游、数字内容等产业集群，争取建立国家级数字出版基地。精心培育文化骨干企业，引导国有和社会资本进入文化产业领域，促进文化企业做强做大。加强版权保护，营造有利于文化发展的环境条件。

　　提升体育事业发展水平。办好“全民健身日”等群众性体育活动，组织好厦门国际马拉松赛十周年系列活动。继续办好海峡杯帆船赛、厦金海峡横渡等涉台大型赛事活动。加大体育设施建设、管理、维护力度，加快市体育中心改造等项目建设。

　　（七）坚持以人为本，做好民生保障工作

　　积极扩大就业。继续落实和完善积极的就业政策，开展各项公共就业服务专项活动，健全工资集体协商、正常增长和支付保障机制。做好大中专毕业生、就业困难对象和零就业家庭等重点群体的就业、再就业工作。争创首批国家创业型城市。

　　健全社会保障体系。以实施《社会保险法》为契机，调整规范社会保障政策，进一步完善基本养老、医疗、工伤、生育、失业保险政策。健全与最低工资标准相挂钩的最低生活保障制度，完善与物价水平相挂钩的困难群众生活补贴标准调整机制。继续提高企业退休金标准和基本医疗保障待遇。全面完成保障性住房建设任务，严把工程质量，做好配售配租工作。进一步完善住房公积金制度。大力发展社会福利事业，完善社会救助体系，支持慈善公益事业和老龄事业，鼓励建设非营利性老年公寓等福利设施，完善社区居家养老服务中心，做好扶老救孤、助残济困工作。尽快建成天马山殡仪馆、市救助管理站、救灾物资储备库。

　　加强和创新社会管理。积极构建符合时代特征、体现厦门特色的社会管理体系。完善社区综合服务管理平台。推行现代化物业管理，创建和谐文明社区。加强村（居）组织换届指导，提升村（居）自治能力和服务水平。大力推进政务诚信、商务诚信、社会诚信建设。大力弘扬互助关爱、见义勇为精神，充分发挥工会、共青团、妇联等群团和民间组织的作用。依法管理宗教事务，发挥宗教团体在经济社会发展中的作用。深化双拥工作，关心支持驻厦部队建设，做好优抚安置工作。

　　扎实推进平安厦门建设。健全社会治安防控体系，排查整治突出治安问题，依法严厉打击各种刑事犯罪。严格落实安全生产责任制，加强重点污染源、重大危险源监管和工业固体、危险废物处置，强化安全管理，避免重大安全事故发生。推进防空防灾一体化建设，强化防灾减灾工作。加强食品药品市场监管，确保市民食品和用药安全。做好突发公共事件应急管理，健全重大事项社会稳定风险评估机制。重视群众信访工作，加强社会矛盾纠纷排查调处。维护劳动者合法权益，构建和谐劳动关系。

　　三、建设廉洁高效的服务型政府

　　我们的政府是人民的政府，必须始终把人民利益放在第一位，坚持以人为本、执政为民，牢固树立为人民服务的宗旨，全面提高服务水平。

　　提高依法行政能力。建设法治政府是维护社会秩序、实现公平正义的重要保障。加强政府法制建设，健全重大行政决策合法性审查、风险评估和公开征求意见制度，切实提高政府科学决策、民主决策和依法决策水平。自觉接受市人大及其常委会的法律监督、工作监督，接受市政协和各民主党派的民主监督。坚持民主集中制，重大事项集体研究决定。加强公务员队伍建设，着力打造一支政治坚定，勇于创新，敢于负责，具有世界眼光和战略思维的公务员队伍。

　　注重反腐倡廉建设。廉洁自律是公务员最基本的要求，廉洁政府是人民群众的殷切期盼。要严格落实《廉政准则》和党风廉政建设责任制，正确行使党和人民赋予的权力，营造风清气正、人和业兴的良好氛围。完善惩治和预防腐败体系，加强工程建设、土地出让、政府采购、产权交易和行政审批等重点领域的制度建设，规范行政权力运行，健全监督制约机制，注重从源头上防范和治理腐败。深化政务公开，逐步扩大公开的领域和范围，增强公共政策制定和公共财政支出的透明度。

　　加强机关作风建设。优良的作风是推动工作落实的重要保证。我们要有“等不起、慢不得、坐不住”的紧迫感，“逆水行舟、不进则退”的危机感，“先行先试、敢为人先”的使命感，不断改进作风，坚持察实情、出实招、办实事、求实效。持续深化效能建设，坚持调查研究，不拍脑袋做决策，改进文风会风。以治庸强素质，以治懒增效率，以治散正风气，健全干部问责追究制度，着力整治不作为、不出力、不落实等问题，把更多的时间、精力用于研究思考大事、谋划长远发展、解决热点难点重点问题。

　　提升为民服务水平。政府工作的出发点和落脚点都是为了实现好、维护好、发展好最广大人民的根本利益。我们要树立为民意识、营造为民氛围、形成为民合力，真诚倾听群众呼声、真实反映群众意愿、真情关心群众疾苦、真心为民排忧解难。强化政府社会管理和公共服务职能，改进政务服务方式，增强服务实效，建设更多便民高效的服务平台，把更多的人力、物力、财力投向民生领域，使特区发展各项成果惠及全体市民。

　　各位代表！蓝图已经绘就，征程已经开启。让我们更加紧密地团结在以胡锦涛同志为总书记的党中央周围，在市委的坚强领导下，进一步解放思想、开拓进取、团结奋斗、真抓实干，加快建设社会主义现代化国际性港口风景旅游城市和海峡西岸重要中心城市，以优异的成绩迎接党的十八大胜利召开！
